# Early renal adaptation after living kidney donation assessed by multiparametric MRI

**DOI:** 10.1186/s12882-026-05509-5

**Published:** 2026-11-14

**Authors:** Eva Carlsson, Per Liss, Jan Weis, Amir Sedigh, Maria K. Svensson

**Affiliations:** 1 Department of Medical Sciences, Renal Medicine, Uppsala University Hospital, Uppsala SE-413 45, Sweden; 2 Department of Surgical Sciences; Radiology, Uppsala University, Uppsala, Sweden; 3 Department of Medical Physics, Uppsala University Hospital, Uppsala, Sweden; 4 Department of Surgical Sciences, Transplantation Surgery, Uppsala University, Uppsala, Sweden; 5 Uppsala Clinical Research Center, Uppsala, Sweden

**Keywords:** Glomerular filtration rate, Kidney volume, Living kidney donor, Multiparametric magnetic resonance imaging, Renal adaptation

## Abstract

**Background:**

Renal adaptation after living kidney donation involves structural, haemodynamic, and functional changes, yet the immediate postoperative phase remains incompletely characterised. We used multiparametric MRI alongside clinical and biochemical assessments to characterise early renal adaptation and its longitudinal change over 12 months, aiming to define the early postoperative imaging phenotype and the course of compensatory adaptation in the remaining kidney.

**Methods:**

In this exploratory longitudinal study, 13 living kidney donors underwent assessment at baseline, postoperative day 3, and at months 3 and 12 after nephrectomy; 12 donors completed follow-up. Multiparametric 3 T MRI measured kidney volume, renal blood flow, perfusion, Apparent Diffusion Coefficient (ADC), T1, and R2*. Kidney function was quantified using iohexol-measured GFR (mGFR). Longitudinal changes were analysed using linear mixed-effects models, and exploratory associations with 12-month mGFR were assessed using linear regression.

**Results:**

Median age was 60 years (IQR 52–65), and 62% were women. Single-kidney GFR (iohexol) increased from a pre-donation (calculated) single-kidney mean GFR value of 51 ± 6 mL/min to 68 ± 9 mL/min at 12 months. Mean kidney volume increased ~ 20%, from 149 ± 24 to 179 ± 21 mL, already by day 3 and reached 186 mL at 12 months. Mean renal blood flow increased ~ 14%, whereas global perfusion decreased ~ 6% at day 3. ADC and cortical T1 increased at day 3 (cortical ADC 2.21 ± 0.12 to 2.35 ± 0.09, medullary ADC 1.86 ± 0.11 to 2.00 ± 0.12 × 10− 3 mm2/s, cortical T1 1199 ± 90 to 1279 ± 51 ms, all *p* ≤ 0.006). These ADC and cortical T1 changes were transient, with values returning towards baseline by month 3. In exploratory regression analyses, higher 12-month mGFR was associated with a smaller early increase in medullary ADC and greater kidney volume at 3 months.

**Conclusions:**

Multiparametric MRI identified a distinct pattern of early renal adaptation following kidney donation, characterised by rapid kidney enlargement, reduced tissue-normalised perfusion, and transient increases in ADC and cortical T1. Exploratory associations between early medullary diffusion changes, kidney volume, and subsequent recovery of kidney function at 12 months are hypothesis-generating and require validation in larger cohorts. These findings support the potential of multiparametric MRI for non-invasive assessment of early renal adaptation.

**Graphical Abstract:**

**Figure:**
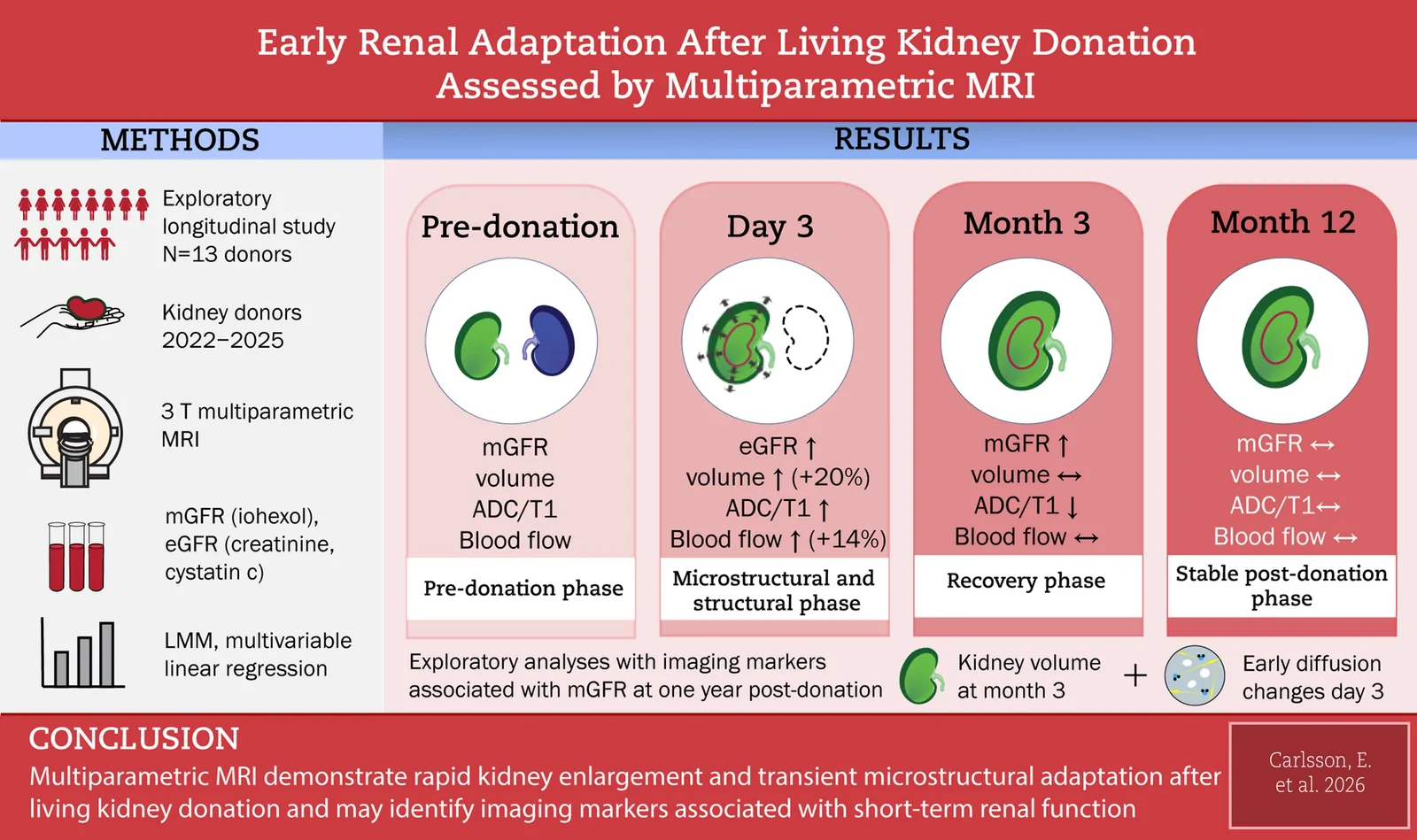

**Supplementary Information:**

The online version contains supplementary material available at https://doi.org/10.1186/s12882-026-05509-5.

## Background

Living donor kidney transplantation is an established treatment for advanced chronic kidney disease. Donation depends on the remaining kidney’s intrinsic adaptive capacity. Following nephrectomy, renal mass is reduced by ~ 50%, with glomerular filtration rate (GFR) recovering to ~ 70% of pre-donation levels [1]. This recovery is largely driven by compensatory hyperfiltration. In healthy donors, hyperfiltration is largely attributed to glomerular hypertrophy and expansion of the filtration surface area [2]. Its predominantly benign long-term trajectory is supported by stable GFR, no excess decline beyond normal ageing, and a low absolute risk of kidney failure [3–6]. Physiological compensation after living kidney donation should be distinguished from maladaptive hyperfiltration described in experimental models and kidney disease [7, 8]. Mechanisms identified in these settings cannot be directly extrapolated to carefully selected healthy donors. The present study focuses on describing early renal adaptation rather than identifying mechanisms of progressive kidney injury.

Because single-nephron function and filtration surface area cannot be measured directly in vivo, renal adaptation is assessed using indirect markers. Multiparametric MRI (mpMRI) provides a non-invasive assessment of renal structure, perfusion, diffusion, and tissue composition within a single examination [9]. Although reproducibility has been demonstrated in healthy individuals [10], clinical translation remains limited by heterogeneous acquisition protocols, lack of standardisation, and incomplete histopathological validation. In contrast to its established use in neurology and oncology [11, 12], renal mpMRI remains largely confined to research settings [9, 13, 14].

The temporal relationship between structural, haemodynamic, and microstructural changes after nephrectomy remains incompletely characterised. Previous donor studies have typically evaluated individual MRI modalities, such as diffusion MRI, ASL perfusion, or BOLD imaging, separately, with few studies integrating these alongside kidney volume, blood flow, perfusion, T1 mapping, and longitudinal measured GFR. Knowledge of the immediate postoperative period is limited because most imaging studies begin after the earliest phase of compensatory change has already occurred. Consequently, it remains unclear how early MRI changes evolve over time and whether they relate to subsequent renal recovery and other outcomes.

We therefore aimed to characterise the early renal adaptation phenotype detected by multiparametric MRI at postoperative day 3 and its evolution over 12 months after living kidney donation. We hypothesised that MRI already on postoperative day 3 would reveal distinct structural, haemodynamic, and microstructural changes reflecting early compensatory adaptation of the remaining kidney and that these changes would evolve in parallel with subsequent functional recovery.

## Methods

### Study design and participants

This exploratory longitudinal cohort study included consecutive living kidney donors undergoing nephrectomy at Uppsala University Hospital (2022–2025). Of 19 consenting donors, 13 were included in the final analysis; 12 completed all four study visits. Six were excluded due to claustrophobia (*n* = 1), acute atrial fibrillation (*n* = 1), postoperative ileus (*n* = 2), or personal reasons (*n* = 2) (Fig. S1).

### Study procedures

Assessments were performed pre-donation, on postoperative day 3, and at 3 and 12 months. Each visit included blood and urine sampling and 3T multiparametric MRI (mpMRI). Kidney function was assessed using creatinine- and cystatin C-based estimated GFR (eGFR), urine albumin-to-creatinine ratio (UACR), and iohexol-measured GFR (mGFR) at baseline and months 3 and 12 [15].

GFR was measured by iohexol clearance both before and after donation. Baseline total mGFR is reported as GFR indexed by body-surface-area (mL/min/1.73 m²) when describing donor characteristics, whereas the longitudinal single-kidney analyses use absolute GFR (mL/min). Before donation, single-kidney GFR was calculated by multiplying total absolute iohexol-measured GFR by the CT-derived split renal function of the kidney remaining after donation.

After donation, absolute iohexol-measured GFR directly reflects the function of the remaining kidney.

$$\eqalign{ & {\rm{Filtration\ fraction }}\left( {{\rm{FF}}} \right){\rm{ was\ calculated\ as\ GFR/}} \cr & \left[ {{\rm{renal\ blood flow\ times }}\left( {{\rm{1}} - {\rm{haematocrit}}} \right)} \right]{\rm{,}} \cr & {\rm{ using\ haematocrit\ measured\ on\ MRI\ day}} \cr}$$

**Fig. 1:**
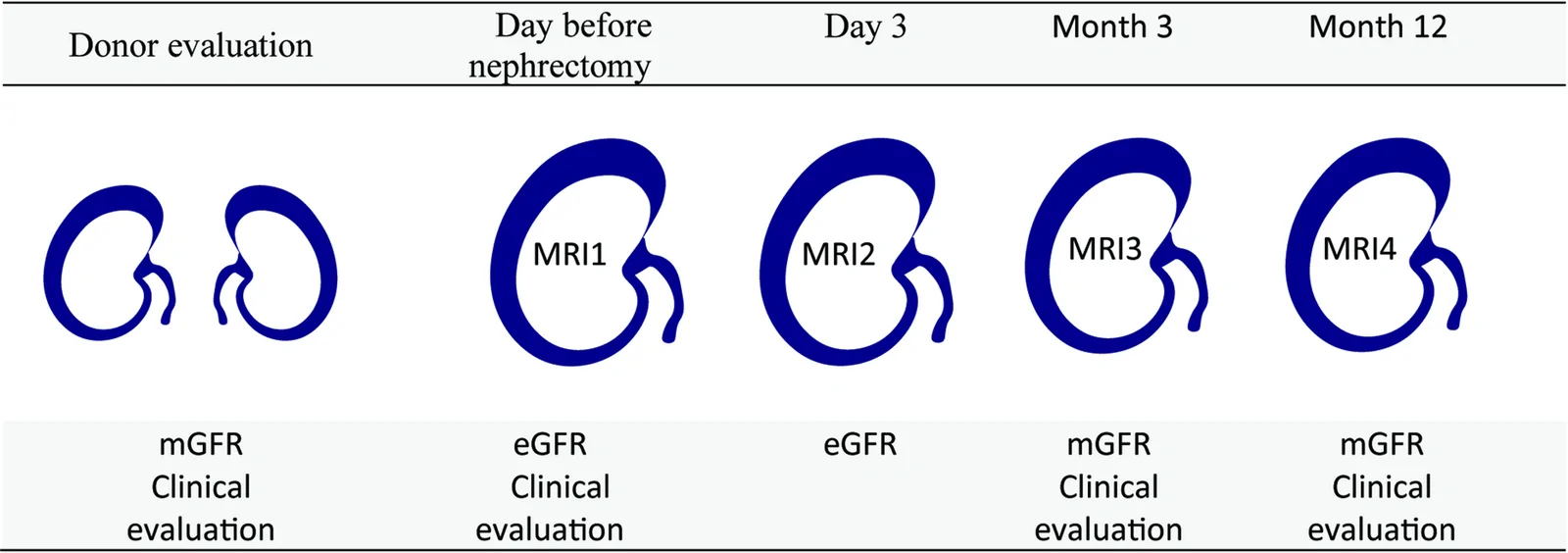
Schematic study protocol

Blood and urine were collected daily during postoperative days 0–4. At each study visit, participants provided three consecutive first-morning urine samples for dipstick analysis and UACR analysis. UACR values below the detection limit (< 3 mg/mmol) were imputed as LOD/√2 and log-transformed. Biobank samples were collected for future analyses. The study protocol is summarised in Fig. 1.

### MRI acquisition and analysis

MRI was performed on a 3T system (Achieva dStream; Philips Healthcare, Best, The Netherlands). Kidney volumes were derived from coronal T2-weighted imaging. Renal blood flow was measured using phase-contrast MRI [16], and the resistive index (RI) was calculated as (peak systolic velocity − end diastolic velocity) / peak systolic velocity.

Global renal perfusion (mL/min/100 g) was calculated as 100 × renal blood flow (mL/min) divided by estimated kidney mass (g). Kidney mass was derived from MRI-measured kidney volume assuming a tissue density of 1.0 g/mL. Cortical perfusionwas assessed separately using arterial spin labelling (ASL) and is reported as cortical blood flow (mL/min/100 g of cortical tissue). Diffusion-weighted imaging provided apparent diffusion coefficient (ADC) values in cortex and medulla. Tissue characteristics were assessed using T1 mapping, and R2* (1/T2*) was used as an indirect marker of tissue oxygenation.

T1 corticomedullary differentiation (CMD) was calculated as cortical T1 minus medullary T1 and analysed using paired t-tests. Dedicated research pulse sequence patches (MR Research Center, Aarhus University Hospital, Denmark) were used for T1 mapping and ASL; these were unavailable in a subset of participants, resulting in missing data. The T1 mapping sequence was based on standard inversion recovery. The perfusion-sensitive ASL sequence used a flow-sensitive alternating inversion-recovery (FAIR) approach [18]. All MRI analyses were performed by a single blinded physicist, following a predefined protocol [17].

### Statistical methods

This was a hypothesis-generating study. Categorical variables are presented as counts (percentages), and continuous variables as means (SD) and medians (IQR).

Longitudinal changes in MRI measurements were analysed using linear mixed-effects models with time as categorical fixed effect, participant-specific random intercept, and unstructured covariance matrix. Models were estimated using restricted maximum likelihood with Satterthwaite approximation. Results are reported as observed means (SD), LMM-adjusted changes, 95% confidence intervals, and p-values. Global time effects were assessed using Type III tests. Sensitivity analyses used paired t-tests or Wilcoxon signed-rank tests. Pairwise comparisons were primarily reported with uncorrected p-values, given the exploratory design of the study and distinct physiological domains assessed. To assess the robustness of the findings to multiple testing, Bonferroni-corrected p-values within each variable and a stricter global Bonferroni correction across all 69 longitudinal pairwise comparisons were calculated and reported for both analyses in the Supplementary Material. Pairwise changes in T1 corticomedullary difference were assessed using two-sided paired t-tests. Both uncorrected p-values and Bonferroni-adjusted p-values for the six comparisons across the four time points were calculated.

Post-hoc exploratory analyses examined associations between MRI-derived variables and 12-month mGFR using multivariable linear regression. Additional exploratory models assessed whether MRI changes provided information beyond established determinants of post-donation kidney function. To minimise overfitting, models were restricted to two variables. Model diagnostics included assessment of collinearity using variance inflation factors (VIF) and leave-one-out sensitivity analyses to evaluate the influence of individual observations. Given the limited sample size, all regression analyses were considered exploratory and hypothesis-generating rather than predictive.

The proportion of missing MRI data ranged from 2.6% to 25.6% and was highest for T1 and perfusion measures. Missing data were handled using all available observations under a missing-at-random assumption. All tests were two-sided, with *p* < 0.05 considered statistically significant. Analyses were performed using IBM SPSS Statistics version 30.0 (IBM Corp., Armonk, NY, USA).

## Results

### Study population and pre-donation characteristics

Thirteen living donors were included; twelve completed all study visits. Baseline characteristics are summarised in Table 1. Median age was 60 years (IQR 52–65), 62% were women, and 54% were genetically related to the recipient. Median pre-donation mGFR was 89 mL/min/1.73 m² (86–94), and median single-kidney GFR (skGFR) was 50 mL/min (47–54). All donors underwent left-sided nephrectomy.

**Table 1:** Pre-donation characteristics of kidney donors

Variables	Kidney donors (*n* = 13)
Age (years)	60 (52–65) 58 (8)
Female/Male n, (%)	8/5 (62/38)
Body mass index (BMI) (kg/m^2^)	26 (24–27) 26 (2)
Body surface area (BSA) (m^2^)	1.85 (1.79–1.96) 1.86 (0.14)
Weight (kg)	76 (71–78) 74 (9
Genetically related to recipient n (%)	7 (54)
Smoker n (%)	1 (8)
Systolic blood pressure (mmHg)	126 (119–142) 130 (15)
Diastolic blood pressure (mmHg)	87 (85–95) 91 (9)
CRP (mg/L)	0.9 (0.49–1.35) 1.4 (2.0)
White blood cell count (WBC) (10^9^/L)	5.1 (4.6–6.1) 5.6 (2.1)
Platelet count (10^9^/L)	255 (216–267) 250 (33)
Sodium (mmol/L)	141 (138–142) 140 (2.7)
Potassium (mmol/L)	4.0 (3.8–4.2) 4.0 (0.2)
Fasting glucose (mmol/L)	5.6 (5.3–5.9) 5.6 (0.5)
Creatinine (µmol/L)	75 (69–83) 77 (11)
eGFR creatinine (mL/min/1.73 m^2^)	76 (66–81) 73 (14)
eGFR Cystatin C (mL/min/1.73m^2^)	84 (79–92) 87 (13)
mGFR iohexol (mL/min/1.73 m^2^)	89 (86–94) 91 (9.2)
Single kidney GFR absolute, remaining kidney (mL/min)	50 (47–54) 51 (6)
Single kidney GFR relative, remaining kidney (mL/min/1.73 m^2^)	47 (44–50) 47 (5)
UACR (mg/mmol)*	0.3 (0.3–0.5) 0.4 (0.2)

Categorical variables are presented as frequency (percentage). Numerical variables are presented as median (Q1 – Q3) and mean (SD). *Mean value of three UACR-values, not log-transformed CRP, C-reactive protein; UACR, urinary albumin to creatinine ratio

### Renal function

Median serum creatinine increased from 75 (69–83) µmol/L pre-donation to 113 (103–134) µmol/L on day 1 and remained at the same level at 12 months. eGFR decreased from 76 (66–81) to 47 (45–55) mL/min/1.73 m² at day 3, with partial recovery but persistent reduction at 12 months. Cystatin C showed a similar trajectory. Calculated pre-donation single-kidney GFR, derived from total iohexol-measured mGFR and CT-based split renal function, was compared with postoperative iohexol-measured mGFR of the remaining kidney. Median single-kidney GFR increased from 50 (47–54) mL/min pre-donation to 66 (58–70) mL/min at month 3 and 68 (61–74) mL/min at month 12. Filtration fraction increased numerically from 0.13 ± 0.02 to 0.15 ± 0.02 at month 3 (*p* = 0.05). Results are presented in Fig. 3a; Tables 2, 3 and 4.

**Table 2:** Pairwise comparisons

Outcome	Time point/contrast	Observed mean (SD)	Adjusted mean change (LMM)	95% CI	P-value	
Kidney function mGFR (mL/min)	Pre-donation	51(6)	reference			
	Month 3	64 (8)	14	11 to 17	**< 0.001**	
	Month 12	68 (9)	17	14 to 20	**< 0.001**	
	Contrast Month 12 vs. Month 3	-	3	0.1 to 6	**0.039**	
Kidney volume (mL)	Pre-donation	149 (24)	reference		-	
	Day 3	179 (21)	31	25 to 37	**< 0.001**	
	Month 3	176 (23)	26	21 to 32	**< 0.001**	
	Month 12	186 (26)	35	28 to 42	**< 0.001**	
	Contrast Month 3 vs. Day 3	-	-5	-10 to 0.3	0.066	
	Contrast Month 12 vs. Month 3	-	8	3 to 14	**0.005**	
Renal artery blood flow (mL/min)	Pre-donation	623 (113)	reference		-	
	Day 3	712 (127)	62	26 to 97	**< 0.001**	
	Month 3	693 (110)	62	11 to 114	**0.017**	
	Month 12	718 (110)	77	32 to 123	**< 0.001**	
	Contrast Month 3 vs. Day 3	-	0.6	-65 to 66	0.99	
	Contrast Month 12 vs. Month 3	-	15	-23 to 53	0.43	
Global perfusion (mL/min/100 g)	Pre-donation	430 (77)	reference		-	
	Day 3	404 (67)	-48	-74 to -22	**< 0.001**	
	Month 3	396 (51)	-36	-69 to -3.0	**0.032**	
	Month 12	389 (63)	-42	-69 to -15	**0.002**	
	Contrast Month 3 vs. Day 3	-	12	-23 to 48	0.50	
	Contrast Month 12 vs. Month 3	-	-6	-29 to 17	0.59	
Perfusion cortex (mL/min/100 g)	Pre-donation	328 (68)	reference		-	
	Day 3	296 (72)	-53	-104 to -2.1	**0.041**	
	Month 3	326 (61)	-10	-45 to 24	0.56	
	Month 12	311 (55)	-24	-51 to 3.9	0.092	
	Contrast Month 3 vs. Day 3	-	43	18 to 67	**< 0.001**	
	Contrast Month 12 vs. Month 3	-	-13	-35 to 8.3	0.23	
Resistive Index	Pre-donation	0.74 (0.05)	reference		-	
	Day 3	0.74 (0.06)	0.00	-0.03 to 0.03	0.92	
	Month 3	0.71 (0.06)	-0.04	-0.07 to 0.00	0.061	
	Month 12	0.71 (0.03)	-0.03	-0.06 to 0.00	**0.028**	
	Contrast Month 3 vs. Day 3	-	-0.04	-0.07 to -0.01	**0.015**	
	Contrast Month 12 vs. Month 3	-	0.00	-0.03 to 0.03	0.93	
R2* cortex (1/s)	Pre-donation	16.4 (0.9)	reference		-	
	Day 3	16.1 (1.0)	-0.48	-0.85 to -0.12	**0.009**	
	Month 3	16.4 (1.0)	-0.10	-0.45 to 0.24	0.55	
	Month 12	16.2 (1.0)	-0.17	-0.52 to 0.19	0.36	
	Contrast Month 3 vs. Day 3	-	0.38	0.05 to 0.71	**0.024**	
	Contrast Month 12 vs. Month 3	-	-0.06	-0.43 to 0.31	0.74	
R* R2* medulla (1/s)	Pre-donation	34.9 (3.4)	reference		-	
	Day 3	32.5 (0.7)	-2.74	-4.92 to -0.56	**0.014**	
	Month 3	32.9 (3.5)	-2.45	-3.91 to -0.99	**0.001**	
	Month 12	32.8 (3.2)	-2.68	-4.36 to -1.01	**0.002**	
	Contrast Month 3 vs. Day 3	-	0.29	-1.03 to 1.61	0.66	
	Contrast Month 12 vs. Month 3	-	-0.23	-1.15 to 0.69	0.62	
ADC cortex (10^− 3^ mm^2^/s)	Pre-donation	2.21 (0.12)	reference		-	
	Day 3	2.35 (0.09)	0.14	0.09 to 0.19	**< 0.001**	
	Month 3	2.28 (0.13)	0.06	-0.01 to 0.14	0.11	
	Month 12	2.26 (0.10)	0.03	-0.04 to 0.10	0.36	
	Contrast Month 3 vs. Day 3	-	-0.08	-0.14 to -0.02	**0.010**	
	Contrast Month 12 vs. Month 3	-	-0.03	-0.08 to 0.02	0.28	
ADC medulla (10^− 3^ mm^2^/s)	Pre-donation	1.86 (0.11)	reference		-	
	Day 3	2.00 (0.12)	0.13	0.07 to 0.18	**< 0.001**	
	Month 3	1.95 (0.15)	0.08	0.02 to 0.14	**0.010**	
	Month 12	1.93 (0.13)	0.05	0.00 to 0.10	0.057	
A	Contrast Month 3 vs. Day 3	-	-0.05	-0.11 to 0.02	0.19	
	Contrast Month 12 vs. Month 3	-	-0.03	-0.10 to 0.04	0.38	
T1 cortex (ms)	Pre-donation	1199 (90)	reference		-	
	Day 3	1279 (51)	62	17.7 to 106.9	**0.006**	
	Month 3	1225 (120)	1.0	-22.5 to 24.5	0.93	
	Month 12	1219 (110)	-22	-68.8 to 24.7	0.35	
Ti	Contrast Month 3 vs. Day 3	-	-61	-100.3 to -22.2	**0.002**	
	Contrast Month 12 vs. Month 3	-	-23	-60.6 to 14.4	0.23	
T1 medulla (ms)	Pre-donation	1748 (173)	reference		-	
	Day 3	1889 (95)	132	38.1 to 225.1	**0.006**	
	Month 3	1847 (246)	87	-10.5 to 184.8	0.080	
	Month 12	1863 (168)	101	58.2 to 143.5	**< 0.001**	
	Contrast Month 3 vs. Day 3	-	-44	-172.1 to 83.2	0.50	
	Contrast Month 12 vs. Month 3	-	14	-68.1 to 95.6	0.74	

Observed values are presented as means (SD). Estimated marginal mean (EMM) changes, additional contrasts, 95% confidence intervals and p-values were obtained from a linear mixed-effects model with time as a fixed effect and participant as a random effect. Pre-donation was used as the reference time point. ADC, apparent diffusion coefficient; R2*, effective transverse relaxation rate; T1, longitudinal relaxation time

**Table 3:** Perioperative biochemical parameters: preoperative, and postoperative days 1–4

	Pre-donation N = 13	Day 1 N = 13	Day 2 N = 13	Day 3 N = 13	Day 4 N = 13
CRP (mg/L)	0.9 (0.5–1.4) 1.4 (2.0)	37 (12–58) 37 (25)	84 (23–120) 76 (60)	56 (21–120) 75 (65)	31 (21–96) 54 (50)
White blood cells (WBC) (10^9^/L)	5.1 (4.6–6.1) 5.6 (2.1)	9.4 (8.7–11.2) 9.9 (2.3)	7.7 (6.8–9.6) 8.5 (2.2)	7.7 (7.0–8.4) 7.7 (1.8)	6.4 (5.7–8.1) 7.1 (2.7)
Platelet count (10^9^/L)	255 (216–267) 250 (33)	202 (174–230) 202 (31)	196 (180–204) 194 (28)	207 (198–249) 218 (45)	232 (222–271) 241 (37)
Sodium (mmol/L)	141 (138–142) 140 (2.7)	137 (133–139) 136 (4.7) [2]	139 (137–141) 138 (2.9)	139 (136–140) 138 (1.9) [1]	138 (137–140) 138 (2.1)
Potassium (mmol/L)	4.0 (3.8–4.2) 4.0 (0.2)	3.9 (3.8–4.2) 4.0 (0.4) [2]	4.0 (3.9–4.2) 4.1 (0.3)	4.2 (3.9–4.4) 4.1 (0.3) [1]	4.2 (4.2–4.4) 4.2 (0.3)
Creatinine (µmol/L)	75 (69–83) 77 (11)	113 (103–134) 118 (21)	116 (103–137) 122 (22)	115 (95–132) 117 (21)	115 (94–127) 116 (23)
eGFR creatinine (mL/min/1.73 m^2^)	76 (66–81) 73 (14)	48 (44–54) 48 (7)	46 (41–54) 46 (8)	47 (45–55) 48 (7)	48 (43–59) 50 (8)
Cystatin C (mg/L)	0.88 (0.82–0.95) 0.88 (0.10)	1.2 (1.1–1.3) 1.2 (0.2) [2]	1.3 (1.1–1.5) 1.3 (0.2) [1]	1.4 (1.2–1.6) 1.4 (0.2) [1]	1.4 (1.3–1.6) 1.4 (0.2)
eGFR Cystatin C (mL/min/1.73 m^2^)	84 (79–92) 87 (13)	58 (54–67) 60 (11) [2]	55 (44–66) 54 (10) [1]	53 (44–60) 52 (8) [1]	51 (43–56) 50 (8)
eGFR mean creatinine/cys C (mL/min/1.73 m^2^)	80 (76–87) 80 (11)	56 (50–58) 54 (8) [2]	51 (45–55) 51 (7) [1]	51 (45–55) 50 (7) [1]	49 (46–57) 50 (7)
U-ACR †	-1.08 (-1.29 –0-79)* -1.04 (0.4)	1.02 (0.35–1.46) 0.82 (0.83)	0.81(-0.01–1.73) 0.45 (0.59)	0.59 (-0.40–1.04) 0.45 (0.81)	-0.01 (-0.85–0.52) -0.01 (1.21)

Numerical variables are presented as median (Q1 – Q3) and mean (SD). Numbers in square brackets indicate number of missing values. † UACR-values are log-transformed. *Mean value of three UACR-values. CRP, C-reactive protein; UACR, urinary albumin to creatinine ratio

**Table 4:** Longitudinal renal function parameters pre-donation, day 3, month 3 and 12 post-donation

	Pre-donation N = 13	Day 3 N = 13	Month 3 N = 13	Month 12 N = 12*
Creatinine (µmol/L)	75 (69–83) 77 (11)	115 (95–132) 117 (21)	112 (95–125) 113 (19)	110 (103–127) 111 (18)
eGFR creatinine (mL/min/1.73 m^2^)	76 (66–81) 73 (14)	47 (45–55) 48 (7)	52 (46–57) 51 (8)	50 (45–61) 52 (10)
Cystatin C	0.88 (0.82–0.95) 0.88 (0.10)	1.4 (1.2–1.6) 1.4 (0.2) [1]	1.3 (1.1–1.3) 1.2 (0.1)	1.2 (1.1–1.3) 1.2 (0.2)
eGFR Cystatin C (mL/min/1.73m^2^)	84 (79–92) 87 (13)	50 (45–59) 51 (8) [1]	56 (54–66) 58 (8)	59 (56–65) 60 (9)
eGFR mean creatinine/cys C (mL/min/1.73 m^2^)	80 (76–87) 80 (11)	51 (45–55) 50 (7) [1]	55 (51–59) 55 (7)	55 (52–60) 56 (9)
Sk mGFR iohexol abs (mL/min)	50 (47–54) 51 (6)	-	66 (58–70) 64 (8)	68 (61–74) 68 (9)
Sk mGFR iohexol rel (mL/min/1.73 m^2^)	47 (44–50) 47 (5)	-	62 (54–65) 60 (7)	61 (59–65) 62 (7)
U-ACR (mg/mmol)†	-1.08 (-1.29 – -0-79) -1.04 (0.4)	0.59 (-0.40 − 1.04) 0.45 (0.81)	-0.99 (-1.30 – -0.33) -0.86 (0.54)	-1.14 (-1.32 – -0.59) -0.99 (0.49)
Filtration fraction	0.12 (0.11–0.14) 0.13 (0.02) [1]	-	0.15 (0.13–0.16) 0.15 (0.02)	-

Numerical variables are presented as median (Q1 – Q3) and mean (SD). Numbers in square brackets indicate number of missing values. *One participant missing at 12-month follow-up. † UACR-values are log-transformed. CRP, C-reactive protein; UACR, urinary albumin to creatinine ratio

### Multiparametric MRI outcomes

**Fig. 2:**
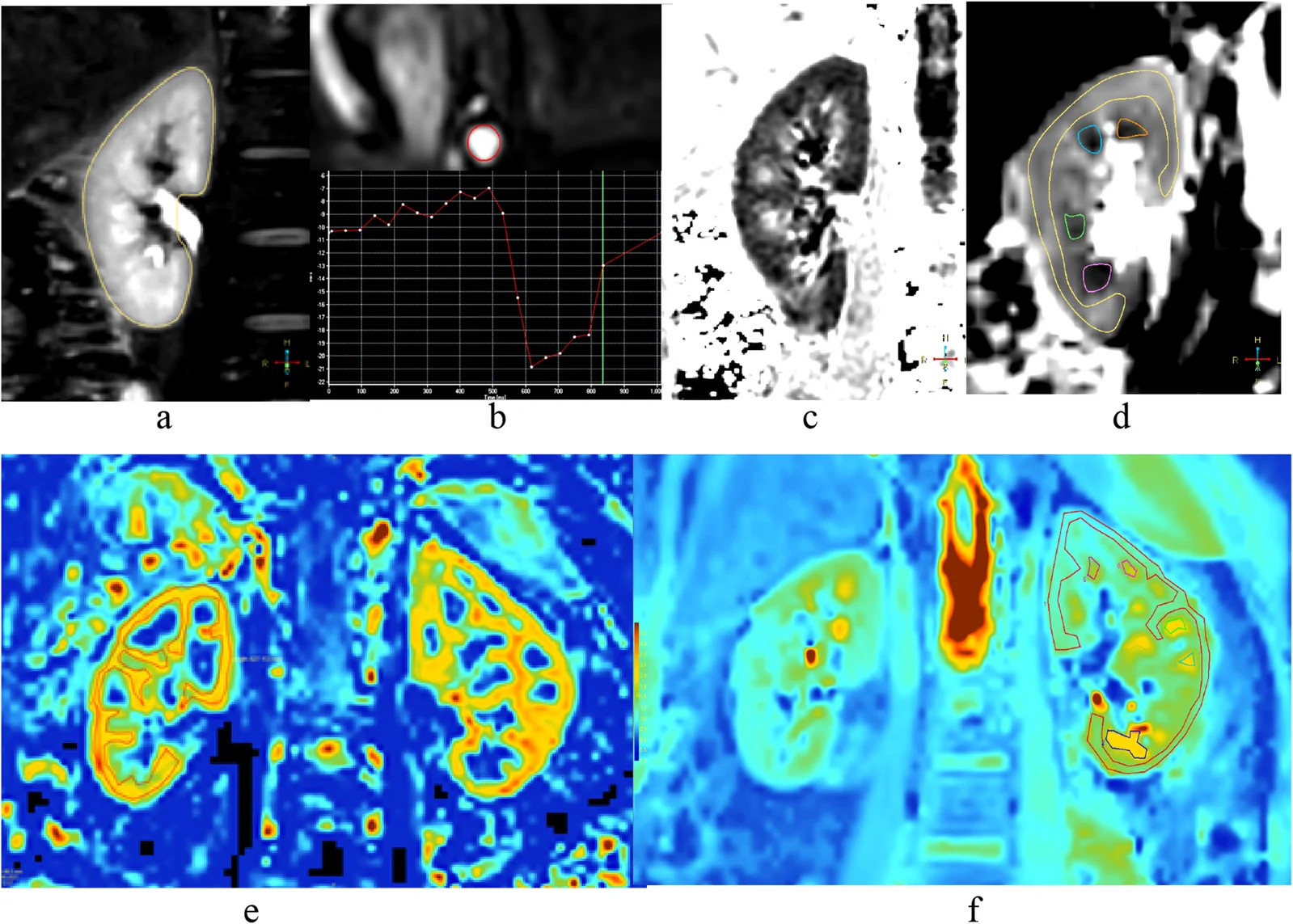
Representative images illustrating the multiparametric renal MRI measurements. (**a**) Anatomical image with kidney segmentation for volume measurement. (**b**) Renal artery blood flow measurement using phase-contrast MRI, with the corresponding flow curve. (**c**) R2* map. (**d**) Apparent diffusion coefficient (ADC) map with cortical and medullary regions of interest. (**e**) Cortical perfusion map obtained using arterial spin labelling. (**f**) T1 map with cortical and medullary regions of interest. Images are provided as illustrative examples. Quantitative results and their units are reported in the tables

Following donation, all MRI-derived variables showed a significant global time effect (*p* < 0.05), although the magnitude and temporal pattern varied. Total MRI acquisition time was approximately 35 min. Data availability differed across modalities due to temporal unavailability of research sequences. Representative images and summary data are shown in Figures 2 and 3b–3d, and Table 2, S1, and S2. Paired-test sensitivity analyses including uncorrected, within-variable Bonferroni-adjusted, and globally Bonferroni-adjusted p-values yielded similar estimates, although significance differed for some comparisons (Table S3).

Corresponding LMM comparisons are presented in Supplementary Table S5.

### Kidney volume changes

Kidney volume increased consistently across all post-donation time points (all *p* < 0.001). Mean volume increased by approximately 20%, from 149 ± 24 mL to 179 ± 21 mL at day 3, corresponding to an adjusted mean increase of 31 mL (95% CI 25–37). Volume remained elevated at month 3 (176 ± 23 mL) and increased by a further 8 mL [3–14] at month 12 (Table 2, S1 and S2, Figs. 2a and 3b).

### Haemodynamic and R2* changes

Mean renal blood flow increased by ~ 14%, from 623 ± 113 pre-donation to 712 ± 127 mL/min at day 3 (adjusted 62 mL/min, 95% CI 26–97), and remained stable at month 12.

In contrast, global and cortical perfusion decreased at day 3 (− 6% and − 10%), corresponding to adjusted reductions of − 48 and − 53 mL/min/100 g. Cortical perfusion partially recovered by 43 mL/min/100 g (95% CI 18–67) by month 3, whereas global perfusion remained reduced at month 12. Resistive index decreased modestly (adjusted − 0.03, 95% CI − 0.06 to 0.00).

Cortical and medullary R2* decreased at day 3 by (− 0.48 and − 2.74 s⁻¹), with partial cortical recovery but persistent medullary reduction at follow-up (Table 2, S1 and S2, Figs. 2b–c and e and 3c).

### MRI-derived tissue characteristics

Both ADC and T1 increased transiently early after nephrectomy. At day 3, cortical and medullary ADC increased by 6% and 8% (adjusted + 0.14 and + 0.13 × 10⁻³ mm²/s). Cortical and medullary T1 increased by 62 ms and 132 ms, respectively. These changes were predominantly transient with cortical ADC and cortical T1 returning towards baseline by month 3 (− 0.08 × 10⁻³ mm²/s and − 61 ms). Medullary changes followed a similar but less consistent pattern (− 0.05 × 10⁻³ mm²/s and − 44 ms). Tissue parameters remained stable thereafter, through month 12 (Table 2, S1 and S2, Figs. 2d and f and 3d).

Among the nine participants with paired pre-donation and month 12 measurements, the mean T1 corticomedullary difference (cortical minus medullary T1) became more negative, from − 524.7 to − 655.2 ms (− 130.6 ms, 95% CI − 177.4 to − 83.7; *p* < 0.001), indicating a greater absolute corticomedullary T1 separation. The difference remained significant after Bonferroni correction for the six pairwise corticomedullary difference (CMD) comparisons (adjusted *p* = 0.0012) (Table 5).

**Table 5:** Observed T1 corticomedullary difference (CMD) and paired changes over time

Time point	*n*	Mean (SD), ms	Median (IQR), ms	
Pre-donation	10	-549 (144)	-559 (-603 – -488)	
Day 3	8	-614 (94)	-602 (-668 – -581)	
Month 3	11	-622 (147)	-620 (-671 – -540)	
Month 12	11	-649 (123)	-647 (-720 – -590)	
**Contrast**	**n paired**	**Mean difference (95% CI)**,** ms**	**Uncorrected p-value**	**Bonferroni-adjusted p-values**
Day 3 - Pre-donation	8	-65 (-193–63)	0.27	1.00
Month 3 - Pre-donation	10	-47 (-127–33)	0.22	1.00
Month 12 - Pre-donation	9	-131 (-177 – -84)	**< 0.001**	**0.0012**
Month 3 - Day 3	8	4 (-126–135)	0.94	1.00
Month 12 - Day 3	7	-50 (-203–103)	0.46	1.00
Month 12 - Month 3	10	-27 (-106–52)	0.46	1.00

CMD was calculated from observed values as cortical T1 minus medullary T1. Pairwise differences are later time point minus earlier time point. Negative values indicate lower cortical than medullary T1. T1, longitudinal relaxation time. Bonferroni-adjusted p-values account for the six pairwise CMD comparisons. The 95% confidence intervals are unadjusted. Statistically significant p-values (*p* < 0.05) are shown in bold

### Associations with 12-month renal function

#### Univariable analyses

Calculated pre-donation single-kidney GFR was strongly associated with 12-month mGFR (B = 1.24, 95% CI 0.59–1.89; *p* = 0.002, R²=0.61). Body surface area and weight were also associated (*p* = 0.012 and *p* = 0.018), whereas no association was observed for height and age. Kidney volume at pre-donation, day 3, and month 3 were associated with 12-month mGFR, with strongest model fit at month 3 (adjusted R²=0.55). No MRI-derived tissue parameters (ADC, T1, or R2*) were significantly associated with 12-month mGFR in univariable analyses (Table S4).

#### Multivariable linear regression

Exploratory models were restricted to two predictor variables. Kidney volume was retained instead of skGFR due to collinearity, and age was excluded as it was not associated with outcome. In the final exploratory model, 3-month kidney volume and the early change in medullary ADC (baseline to day 3) explained 81% of the observed variance in 12-month mGFR. In the corresponding model, the change in cortical ADC was not significantly associated with 12-month mGFR after adjustment for kidney volume at month 3 (Supplementary Table S6). Larger kidney volume was positively associated (B = 0.39 per mL, *p* < 0.001), whereas a greater increase in medullary ADC was inversely associated with 12-month mGFR (B = − 44.93, 95% CI − 88.09 to − 1.76; *p* = 0.043) (Table 6). Collinearity was minimal (VIF = 1.02 for both variables). In leave-one-out analyses, the association with kidney volume remained robust, whereas the association with medullary ADC remained directionally consistent but varied in statistical significance. Given that the regression model included only 11 participants, results should be considered exploratory and interpreted with caution. Detailed model diagnostics are provided in Supplementary Table S7.

**Table 6:** Multivariable linear regression models for factors associated with 12-month mGFR

Model and variables	Β (95% CI)	p-value	Adj. R2
**Model 1** (*n* = 12)			
Baseline skmGFR (mL/min)	1.24 (0.59–1.89)	**0.002**	0.61
**Model 2** (*medullary ADC, n = 11*)			
Kidney volume at month 3 (mL)	0.39 (0.25–0.52)	**< 0.001**	0.81
∆ *Medullary* ADC (pre-donation to day 3) (10^− 3^ mm^2^/s)	-44.93 (-88.09 – -1.76)	**0.043**	

B values are regression coefficients with 95% confidence intervals (95% CI). ADC, apparent diffusion coefficient

## Discussion

In this exploratory longitudinal study of living kidney donors, the principal finding was that postoperative day 3 mpMRI identified a distinct early adaptation phase characterised by rapid kidney enlargement, reduced tissue-normalised perfusion, and transient diffusion and T1 changes. These early imaging alterations preceded subsequent stabilisation of single-kidney GFR, supporting a temporal model in which acute and early structural and tissue-level responses are followed by sustained functional compensation during the first year after donation. Increases in kidney volume and single-kidney GFR after donation are expected. The contribution of this study is the combined assessment of multiple MRI-derived measures during the immediate postoperative phase and their differing trajectories over follow-up. The early haemodynamic, diffusion, and cortical T1 changes describe this adaptation in more detail but do not establish injury or specific underlying biological mechanisms.

Kidney volume increased by approximately 20% within three days and remained elevated at 12 months. This demonstrates that kidney enlargement is detectable in the immediate postoperative period but does not establish the underlying cause of the enlargement. Postoperative oedema, changes in renal blood volume (increased by 14%), tubular expansion, and perioperative fluid shifts may contribute to the observed increase in kidney size. Our measurements cannot distinguish these processes from structural tissue growth or determine their relative contributions. However, the observation of enlargement as early as postoperative day 3 is noteworthy and suggests that renal adaptation may commence very shortly after nephrectomy.

Experimental studies demonstrate that hypertrophic signalling pathways are activated within hours after nephrectomy, leading to enlargement of tubular cells, and to a limited collecting-duct hyperplasia [18], whereas the increase in kidney volume at later time points is compatible with compensatory hypertrophy, although this interpretation remains indirect. The observed volumetric enlargement is consistent with previous imaging studies [19–21] and was accompanied by a 28% increase in mean single-kidney GFR at month 3 and a 34% increase at month 12, in line with prior reports of compensatory functional adaptation after nephrectomy [22–25].

Haemodynamic adaptation was less pronounced than structural and functional changes. Renal blood flow increased after donation, whereas global tissue-normalised perfusion (mL/min/100 g kidney tissue) decreased, indicating that blood flow did not fully match the increase in kidney mass. Because global perfusion was derived from blood flow divided by estimated kidney mass, the decline may partly reflect disproportionate kidney growth rather than a true reduction in tissue perfusion. Therefore, these findings should not be interpreted as direct evidence of impaired microvascular perfusion or oxygen delivery.

Cortical perfusion, assessed separately using ASL, also decreased early and partially recovered by month 3, while global perfusion remained reduced, indicating that absolute blood flow and tissue-normalised perfusion may reflect different physiological aspects of adaptation. Together with increased mGFR and filtration fraction, these findings could support the concept that hyperfiltration primarily reflects intraglomerular haemodynamic adjustments increasing filtration efficiency rather than increased tissue perfusion alone. However, though these findings describe postoperative haemodynamic adaptation, they do not establish the underlying intraglomerular mechanisms.

R2* decreased in both the cortex and medulla, but the reduction was transient in the cortex and sustained in the medulla. This dissociation may reflect redistribution of oxygen supply and demand, potentially driven by altered tubular workload or altered microvascular organisation rather than by perfusion alone. However, these changes cannot be attributed specifically to altered tissue oxygenation, because R2* is influenced by blood volume, haematocrit, hydration status, and tissue composition, and remains an indirect oxygenation marker [26, 27].

Transient increases in cortical and medullary ADC and cortical T1 at day 3 indicate early non-specific tissue-level responses. These changes largely normalised by month 3, suggesting reversibility. Potential mechanisms include altered tubular transport, interstitial fluid shifts, or changes in water mobility, though these parameters lack biological specificity. The observed changes therefore cannot distinguish structural adaptation from acute postoperative fluid shifts. Although a previous study found no significant effect of oral hydration on ADC in healthy individuals [28], this does not exclude contributions from perioperative fluid changes in our cohort. The ADC values in our cohort were similar to previously reported values, although medullary values were slightly lower. Direct comparisons are limited by differences in the study protocols and populations [29, 30]. The corticomedullary T1 difference became more negative at month 12, indicating increased cortical–medullary separation. While reduced corticomedullary differentiation has been described in kidney injury [31], the significance of increased T1 separation after donor nephrectomy remains uncertain.

In exploratory analyses, kidney volume at 3 months and early medullary ADC change were jointly associated with 12-month mGFR. Kidney volume at month 3 may reflect longer-term adaptation, whereas early changes in ADC may capture acute tissue responses following donation. However, neither measure provides direct insight into the underlying biological mechanisms. For a given kidney volume, a larger increase in medullary ADC at day 3 was associated with lower 12-month mGFR, potentially reflecting transient fluid shifts rather than more effective adaptation processes. These findings are hypothesis-generating and should not be interpreted as evidence of causality or prognostic utility. Validation in larger, independent cohorts is required before any prognostic interpretation can be inferred.

### Strengths and limitations

The strengths of this study include the prospective longitudinal design, fixed early postoperative assessment, 12-month follow-up, and integration of mpMRI with repeated iohexol-measured GFR, enabling a comprehensive assessment of structural, haemodynamic, and tissue-level changes. Mixed-effects models allowed efficient use of all repeated observations and handling of missing data.

Limitations include small sample size, limiting statistical power and stability of the estimates. The large number of outcomes and comparisons also increases the risk of type I error. Primary analyses used uncorrected p-values. Within-variable and global Bonferroni corrections were reported as sensitivity analyses; findings that did not remain significant after correction require cautious interpretation. Some MRI data were missing because the required sequences, particularly those for T1 mapping and ASL, were temporarily unavailable.

Moreover, the ASL technique used in this study did not permit measurement of medullary perfusion. MRI findings were not validated against histology, and interpretation of ADC, T1, and R2* remains indirect. Kidney volume measurements likewise cannot distinguish structural hypertrophy from postoperative oedema or changes in vascular and tubular compartment volumes, particularly in the immediate postoperative period. In addition, global perfusion was normalised to estimated kidney mass and does not account for relative contributions of intravascular or tubular luminal volume fractions [32]. As a result, changes in kidney composition after donation may have influenced perfusion estimates and could also confound the interpretation of R2* measurements. Finally, the healthy donor cohort limits generalisability, particularly to individuals with lower baseline kidney function or comorbidity. The sample size and 12-month follow-up do not permit assessment of long-term kidney disease risk or validation of prognostic biomarkers. Longer follow-up is needed to determine whether early MRI-derived changes are associated with long-term renal outcomes.

### Interpretation and implications

These findings extend previous work by demonstrating a temporal sequence of early post-donation renal adaptation with early kidney enlargement and transient tissue changes followed by persistently elevated kidney volume and increased single-kidney GFR at later follow-up. The divergence between renal blood flow, perfusion, filtration fraction, and mGFR indicates that structural, haemodynamic, and functional adaptations are not proportionally coupled. Given the exploratory nature of the study and the number of pairwise comparisons, we interpreted the analyses with caution. Several comparisons were significant in uncorrected analyses but did not remain significant after Bonferroni correction, particularly for RI, R2*, and some perfusion and T1 comparisons. In contrast, increases in kidney volume, single-kidney GFR, early changes in ADC, and selected day 3 haemodynamic changes remained significant after correction. However, considering the size of the cohort, the findings are best viewed as hypothesis-generating.

Multiparametric MRI revealed variation in the magnitude and timing of post-donation changes, but whether this variation reflects differences in adaptive capacity or has prognostic significance remains unknown. Future studies should prospectively validate the early postoperative MRI phenotype and determine whether acute imaging changes provide mechanistic or clinical value beyond established functional measures. Future studies could incorporate measurements of tubular volume fraction (TVF) and blood volume fraction (BVF) to investigate how changes in renal compartments contribute to post-donation kidney enlargement. Such measurements could enable evaluation of perfusion normalised to an estimated tissue compartment excluding blood and tubular luminal volumes and help distinguish blood-volume-related effects on R2* from changes in oxygenation. Integration with circulating blood and urinary biomarkers may further improve our understanding of the biological processes driving renal adaptation and compensation after living kidney donation.

## Conclusion

In this exploratory cohort, repeated multiparametric MRI demonstrated a distinct pattern of early renal adaptation following living kidney donation, characterised by rapid enlargement in kidney volume, reduced tissue-normalised perfusion despite an increase in blood flow, and transient changes in diffusion and cortical T1. Repeated assessments, including imaging on postoperative day 3, were feasible in this clinical study setting, allowing non-invasive characterisation of renal adaptation and its longitudinal trajectory. Exploratory associations between early medullary diffusion changes, kidney growth, and 12-month kidney function are hypothesis-generating and require confirmation in larger, independent cohorts. These findings suggest that multiparametric MRI may provide a feasible non-invasive approach for assessing early renal adaptation after living kidney donation, although its clinical and prognostic utility remains to be established.

**Fig. 3a:**
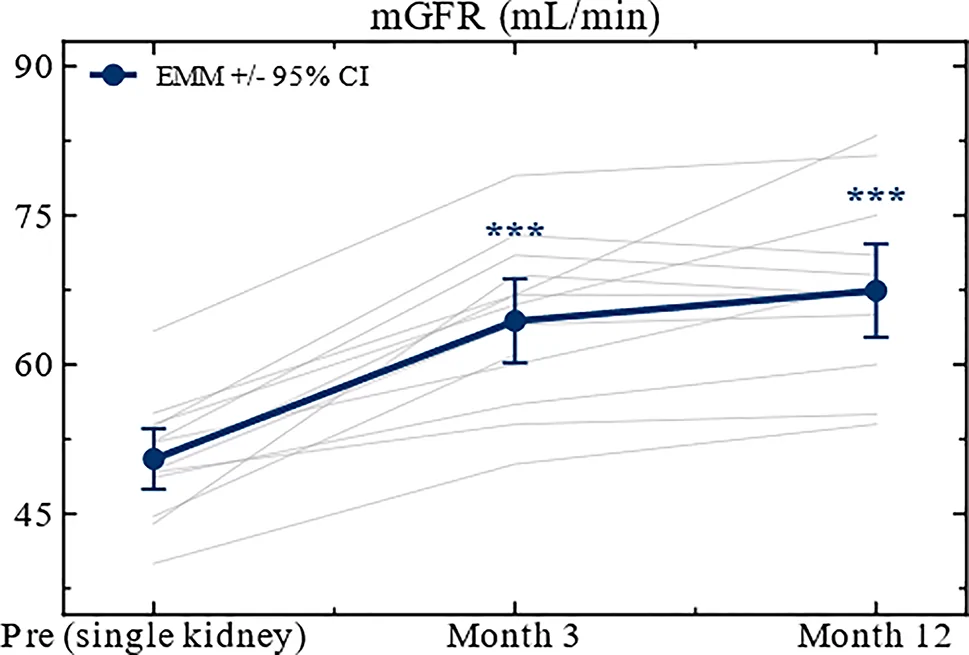
Longitudinal change in single-kidney glomerular filtration rate following donor nephrectomy. Gray lines represent individual participant trajectories. Blue symbols and error bars represent estimated marginal means and 95% confidence intervals from the linear mixed-effects model. Measured GFR increased significantly from the calculated pre-donation single-kidney value at month 3 and remained elevated at month 12. Pre-donation single-kidney GFR was calculated from total iohexol-measured GFR and CT-derived split renal function. Postoperative mGFR was measured by iohexol clearance in the remaining kidney. ***p < 0.001 versus pre-donation

**Fig. 3b:**
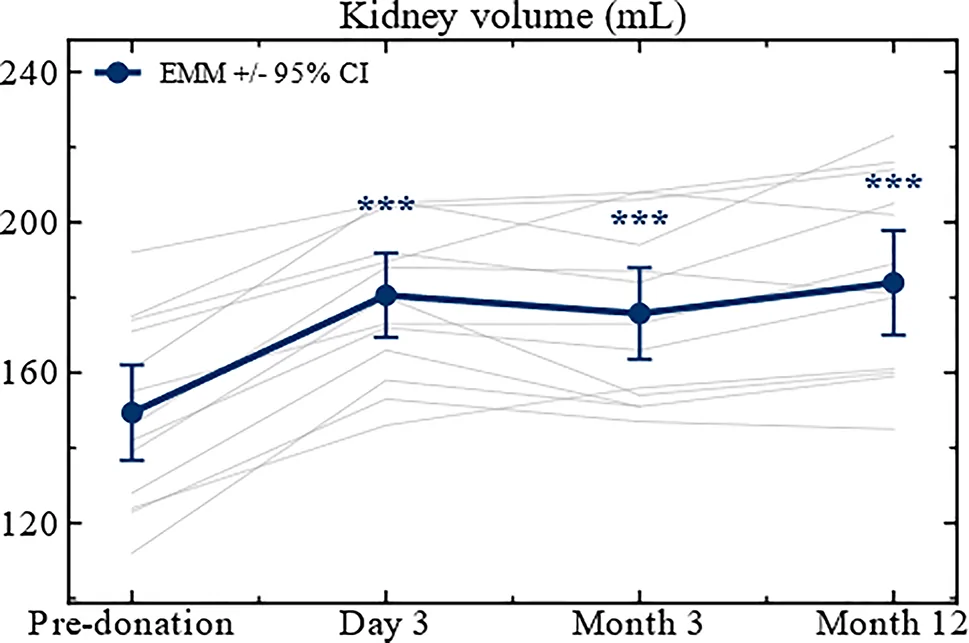
Longitudinal change in kidney volume following living kidney donation. Grey lines represent individual participant trajectories. Blue symbols and error bars represent estimated marginal means and 95% confidence intervals from the linear mixed-effects model. Kidney volume increased significantly by postoperative day 3 and remained elevated at months 3 and 12 compared with pre-donation. ***p < 0.001 versus pre-donation

**Fig. 3c:**
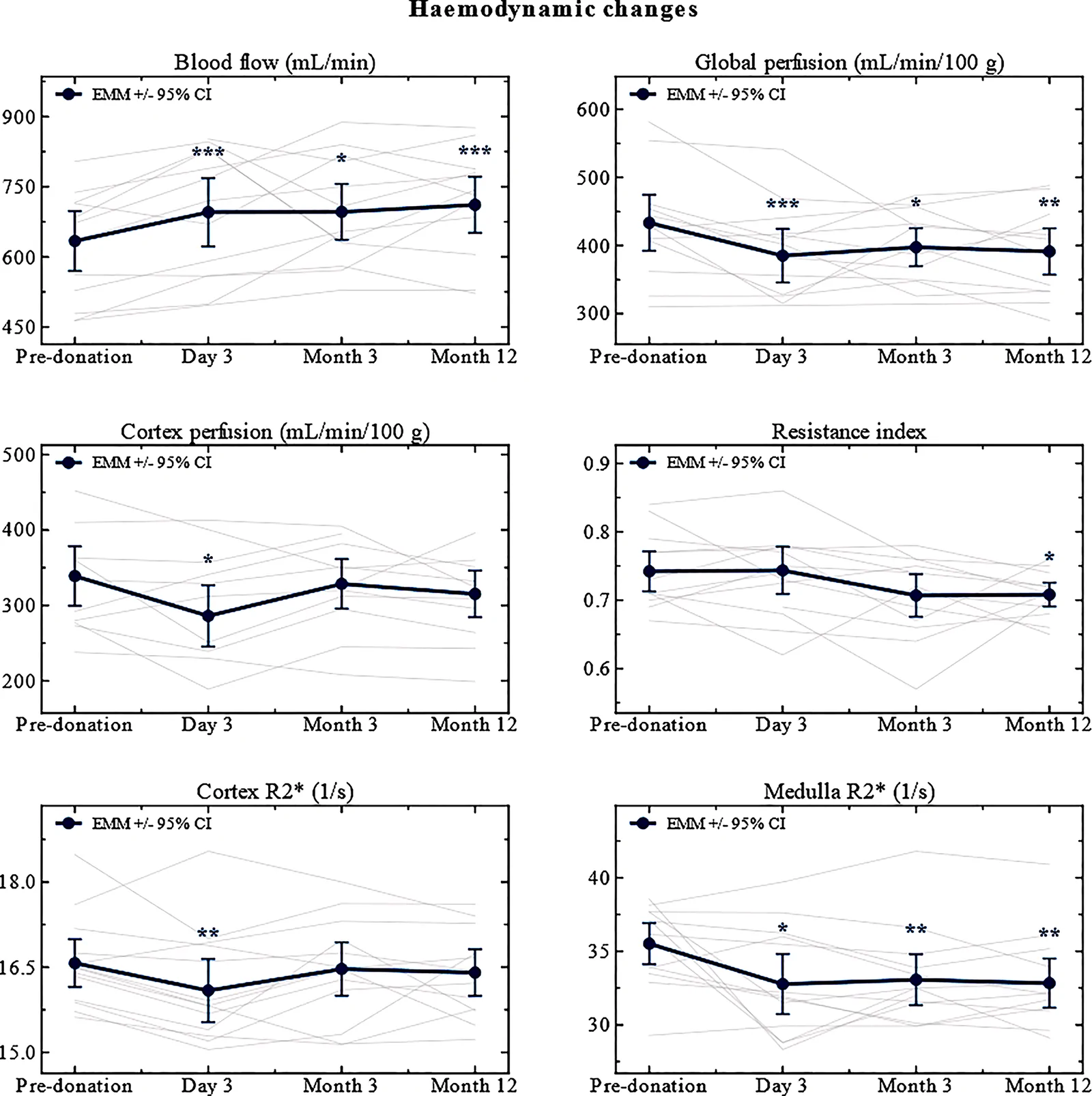
Longitudinal haemodynamic and R2* changes following living kidney donation. Grey lines represent individual participant trajectories. Blue symbols and error bars represent estimated marginal means with 95% confidence intervals from the linear mixed-effects models. Renal blood flow increased after donation and remained elevated throughout follow-up. Global perfusion decreased at postoperative day 3 and remained below pre-donation levels, whereas cortical perfusion decreased transiently at day 3 and subsequently returned towards baseline. The resistive index showed a modest reduction during follow-up. Cortical R2* decreased transiently at day 3, while medullary R2* remained reduced at months 3 and 12. *p < 0.05, **p < 0.01, and ***p < 0.001 versus pre-donation

**Fig. 3d:**
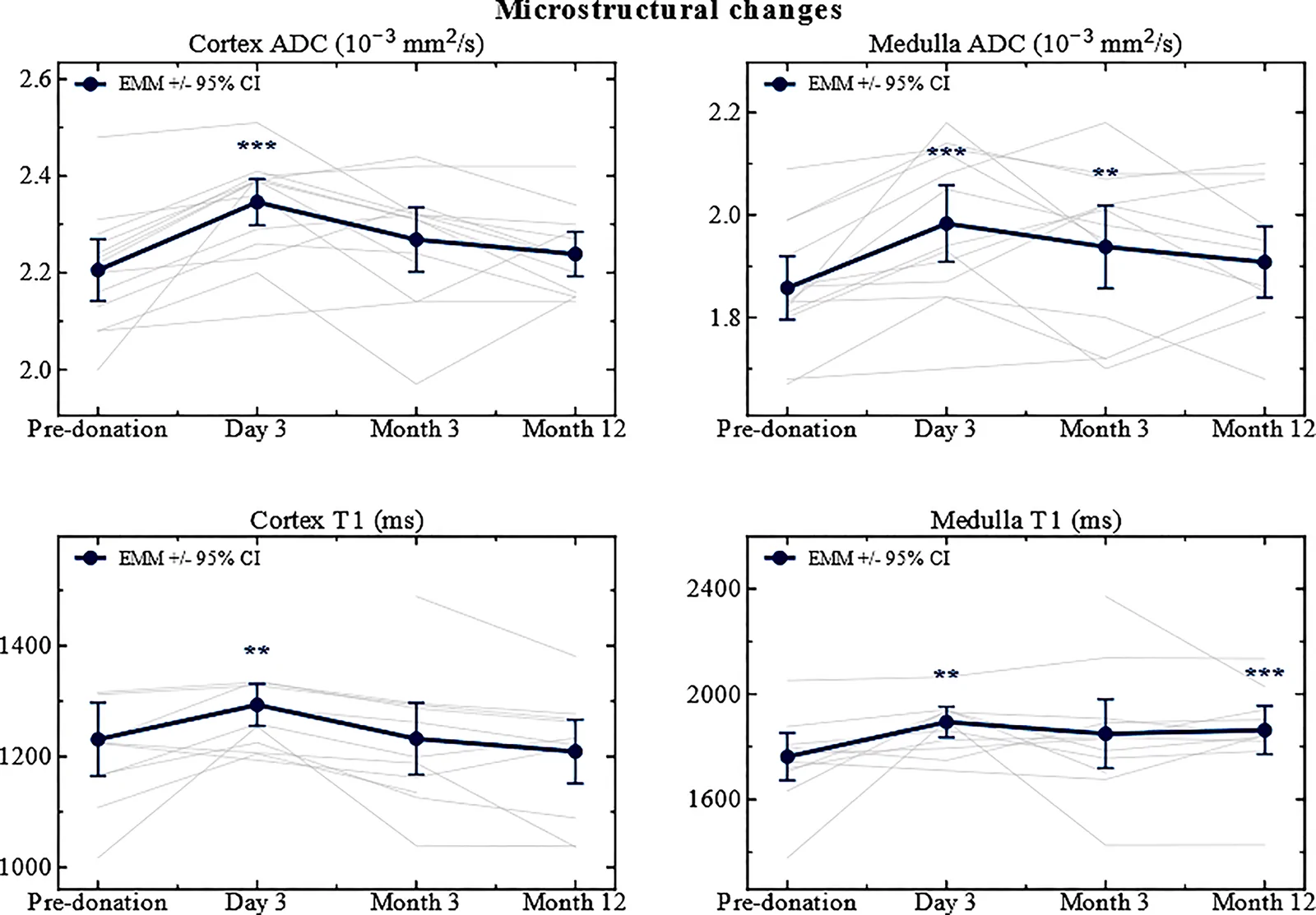
Longitudinal changes in renal diffusion and T1 following living kidney donation. Grey lines represent individual participant trajectories. Blue symbols and error bars represent estimated marginal means with 95% confidence intervals from the linear mixed-effects models. Cortical and medullary ADC increased at postoperative day 3 and subsequently decreased towards pre-donation levels. Cortical T1 showed a similar transient increase at day 3, whereas medullary T1 increased early and remained modestly elevated during follow-up. *p < 0.05, **p < 0.01, and ***p < 0.001 versus pre-donation

## Supplementary Information

Below is the link to the electronic supplementary material.

Supplementary Material 1

## Data Availability

The datasets used and/or analysed during the current study are available from the corresponding author on reasonable request.

## Abbreviations

ADC: Apparent Diffusion Coefficient
ASL: Arterial Spin Labelling
BCM: Body Composition Monitoring
BMI: Body Mass Index
BOLD: Blood Oxygen Level-Dependent
BSA: Body Surface Area
CI: Confidence Interval
CKD: Chronic Kidney Disease
CMD: Corticomedullary Differentiation
CT: Computed Tomography
eGFR: Estimated Glomerular Filtration Rate
EMM: Estimated Marginal Means
FAIR: Flow-Sensitive Alternating Inversion Recovery
FF: Filtration Fraction
IQR: Interquartile Range
LMM: Linear Mixed-Effects Model
LOD: Limit of Detection
mGFR: Measured Glomerular Filtration Rate
mpMRI: Multiparametric Magnetic Resonance Imaging
MRI: Magnetic Resonance Imaging
R2*: Effective Transverse Relaxation Rate (1/T2*)
RI: Resistive Index
SD: Standard Deviation
skGFR: Single-Kidney Glomerular Filtration Rate
T1: Longitudinal Relaxation Time
T2: Transverse Relaxation Time
T2*: Effective Transverse Relaxation Time
UACR: Urinary Albumin-to-Creatinine Ratio
VIF: Variance Inflation Factor
WBC: White Blood Cell Count
