# Supplementary material for: Early renal adaptation after living kidney donation assessed by multiparametric MRI
Source: BMC Nephrol. 2026 Nov 14;27:678. doi: 10.1186/s12882-026-05509-5 (PMC13769189; doi:10.1186/s12882-026-05509-5)
Supplement: Supplementary file 1 [file 12882_2026_5509_MOESM1_ESM.docx]

**ADDITIONAL MATERIALS**

| **Additional fig./table** | **Title** |  |
| --- | --- | --- |
| Fig. S1 | Study flow chart of donor inclusion, exclusions, and follow-up |  |
| Table S1 | Observed MRI-derived measures across study visits |  |
| Table S2 | Linear mixed-effects model estimates for MRI-derived measures |  |
| Table S3 | Bonferroni-corrected pairwise comparisons using paired t-test or Wilcoxon signed-rank test |  |
| Table S4 | Exploratory univariable associations with 12-month measured GFR |  |

Table S5	Bonferroni-corrected linear-mixed model (LMM) pairwise comparisons

Table S6	Multivariable linear regression models for factors associated with 12-month mGFR

Table S7	Compact diagnostics for the exploratory multivariable regression model with 12-month measured GFR as outcome.

**FIGURE S1**

**Fig S1. Participant flowchart.** Nineteen donors consented, 18 completed baseline MRI, and 13 were included in the final analysis.

**TABLE S1**

**MRI parameter summary statistics and missing data**

|  | Pre-donation  (n=13) | Day 3 postoperative  (n=13) | Month 3  (n=13) | Month 12  (n=12)* |
| --- | --- | --- | --- | --- |
| Kidney volume  (mL) | 146 (126 – 173)  149 (24) | 177 (160 – 201)  179 (21) [1] | 173 (153 – 200)   176 (23) | 185 (160 – 211)  186 (26) |
| Renal artery blood flow  (mL/min) | 642 (504 – 715)  623 (113) [1] | 720 (560 – 829)  712 (127) [3] | 657 (618 – 807)  693 (110) | 732 (645 – 784)  718 (110) |
| Global perfusion  (mL/min/100 g) | 429 (385 – 458)  430 (77) [1] | 409 (328 – 434)  404 (67) [3] | 392 (350 – 438)  396 (51) | 394 (333 – 436)  389 (63) |
| Perfusion cortex  (mL/min/100 g) | 313 (276 – 375)  328 (68) [3] | 312 (235 – 349)  296 (72) [4] | 323 (285 – 382)  326 (61) [2] | 317 (272 – 351)  311 (55) [1] |
| Resistive Index (RI) | 0.73 (0.71 – 0.78)  0.74 (0.05) [1] | 0.74 (0.69 – 0.78)  0.74 (0.06) [3] | 0.73 (0.67 – 0.76)  0.71 (0.06) | 0.71 (0.68 – 0.73)  0.71 (0.03) |
| Cortex R2* (1/s) | 16.4 (15.8 – 16.9)  16.4 (0.91) | 15.8 (15.3–16.8)  16.1 (1.0) [2] | 16.5 (15.3–17.1)  16.4 (1.0) | 16.2 (15.6–17.0)  16.2 (1.0) |
| Medulla R2* (1/s) | 36.1 (33.3–37.7)  34.9 (3.4) | 31.7 (29.0 – 36.2)  32.5 (0.68) [2] | 32.5 (30.0 – 34.3)  32.9 (3.45) [1] | 32.1 (30.6 – 34.6)  32.8 (3.2) |
| Cortex ADC  (10^-3^ mm^2^/s) | 2.23 (2.12 – 2.28)  2.21 (0.12) | 2.39 (2.28 – 2.40)  2.35 (0.09) [1] | 2.32 (2.20 – 2.35)  2.28 (0.13) | 2.23 (2.16 – 2.32)  2.26 (0.10) |
| Medulla ADC  (10^-3^ mm^2^/s) | 1.85 (1.81 – 1.94)  1.86 (0.11) | 2.00 (1.89 – 2.13)  2.00 (0.12) [1] | 1.99 (1.78 – 2.05)  1.95 (0.15) | 1.94 (1.85 – 2.06)  1.93 (0.13) [1] |
| Cortex T1 (ms) | 1224 (1150 – 1251)  1199 (90) [3] | 1274 (1232 – 1333)  1279 (51) [5] | 1199 (1135 – 1293)  1225(120) [2] | 1248 (1121 – 1288)  1219 (110) [1] |
| Medulla T1 (ms) | 1758 (1687 – 1825)  1748 (173) [3] | 1899 (1810 – 1939)  1889 (95) [4] | 1831 (1701 – 1937)  1847 (246) [2] | 1866 (1825 – 1932)  1863 (168) [1] |

Variables are presented as median (Q1 – Q3) and mean (SD). Numbers in square brackets indicate number of missing values. *One participant missing at 12-month follow-up.

**TABLE S2**

**Global time effects and longitudinal changes assessed using linear mixed-effects models**

| Outcome |  | | | | p-value | |
| --- | --- | --- | --- | --- | --- | --- |
| Kidney volume (mL) |  | | | | **<0.001** | |
| Blood flow (mL/min) |  | | | | **<0.001** | |
| Global perfusion (mL/min/100 g) |  | | | | **<0.001** | |
| Perfusion cortex (mL/min/100 g) |  | | | | **<0.001** | |
| RI |  | | | | **0.007** | |
| R2*cortex (1/s) | |  | | | **0.046** | |
| R* R2* medulla (1/s) | | |  | | **0.008** | |
| ADC cortex (10^-3^ mm^2^/s) | | | | **<0.001** | |  |
| ADC medulla  (10^-3^mm^2^/s) | | |  | | **<0.001** | |
| T1 cortex (ms) |  | | | | **<0.001** | |
| T1 medulla (ms) |  | | | | **<0.001** | |

P-values for time effects were obtained from linear mixed-effects models.

**TABLE S3**

**Bonferroni-corrected pairwise comparisons using paired t-test or Wilcoxon signed-rank test**

| **Variable** | **Time point** | **n** | **Test** | **Change** | **95% CI** | **p value** | **Bonferroni p value** | **Global Bonferroni p value** |
| --- | --- | --- | --- | --- | --- | --- | --- | --- |
| mGFR (mL/min) | Month 3 vs Pre-donation | 13 | Paired t-test | 13.87 | 10.48 to 17.26 | **<0.001** | **<0.001** | **<0.001** |
| mGFR (mL/min) | Month 12 vs Pre-donation | 12 | Paired t-test | 16.92 | 13.39 to 20.45 | **<0.001** | **<0.001** | **<0.001** |
| mGFR (mL/min) | Month 12 vs Month 3 | 12 | Paired t-test | 3.25 | -0.20 to 6.70 | 0.062 | 0.187 | 1.000 |
| Kidney volume (mL) | Day 3 vs Pre-donation | 12 | Paired t-test | 31.00 | 23.73 to 38.27 | **<0.001** | **<0.001** | **<0.001** |
| Kidney volume (mL) | Month 3 vs Pre-donation | 13 | Paired t-test | 26.38 | 20.39 to 32.38 | **<0.001** | **<0.001** | **<0.001** |
| Kidney volume (mL) | Month 12 vs Pre-donation | 12 | Paired t-test | 33.75 | 25.48 to 42.02 | **<0.001** | **<0.001** | **<0.001** |
| Kidney volume (mL) | Month 3 vs Day 3 | 12 | Paired t-test | -5.50 | -11.50 to 0.50 | 0.069 | 0.412 | 1.000 |
| Kidney volume (mL) | Month 12 vs Day 3 | 11 | Paired t-test | 3.09 | -5.32 to 11.51 | 0.432 | 1.000 | 1.000 |
| Kidney volume (mL) | Month 12 vs Month 3 | 12 | Paired t-test | 8.42 | 1.75 to 15.09 | **0.018** | 0.108 | 1.000 |
| Blood flow (mL/min) | Day 3 vs Pre-donation | 9 | Paired t-test | 60.89 | 14.03 to 107.8 | **0.017** | 0.103 | 1.000 |
| Blood flow (mL/min) | Month 3 vs Pre-donation | 12 | Paired t-test | 65.50 | 1.799 to 129.2 | **0.045** | 0.269 | 1.000 |
| Blood flow (mL/min) | Month 12 vs Pre-donation | 11 | Paired t-test | 70.18 | 12.37 to 128.0 | **0.022** | 0.133 | 1.000 |
| Blood flow (mL/min) | Month 3 vs Day 3 | 10 | Paired t-test | -10.30 | -105.1 to 84.47 | 0.811 | 1.000 | 1.000 |
| Blood flow (mL/min) | Month 12 vs Day 3 | 9 | Paired t-test | -2.22 | -91.24 to 86.79 | 0.956 | 1.000 | 1.000 |
| Blood flow (mL/min) | Month 12 vs Month 3 | 12 | Paired t-test | 17.58 | -27.23 to 62.40 | 0.406 | 1.000 | 1.000 |

Differences are later minus earlier time point. Change is the mean paired difference for paired t-tests and the median paired difference for Wilcoxon signed-rank tests. Confidence intervals are not adjusted for multiplicity. Tests are two-sided. Bonferroni p = min(p × m, 1), where m = 3 for mGFR and 6 for each MRI variable. Global Bonferroni p = min(p × 69, 1), across all comparisons in this table. Bold indicates p < 0.05 in the respective column. Pre-donation mGFR refers to calculated single-kidney GFR; postoperative mGFR was measured by iohexol clearance. n denotes the number of paired observations.

**TABLE S3 continued**

| **Variable** | **Time point** | **n** | **Test** | **Change** | **95% CI** | **p value** | **Bonferroni p value** | **Global Bonferroni p value** |
| --- | --- | --- | --- | --- | --- | --- | --- | --- |
| Global perfusion (mL/min/100 g) | Day 3 vs Pre-donation | 9 | Paired t-test | -52.89 | -87.62 to -18.16 | **0.008** | **0.048** | 0.548 |
| Global perfusion (mL/min/100 g) | Month 3 vs Pre-donation | 12 | Wilcoxon | -5.00 | -102.5 to 17.50 | 0.401 | 1.000 | 1.000 |
| Global perfusion (mL/min/100 g) | Month 12 vs Pre-donation | 11 | Paired t-test | -44.73 | -79.30 to -10.16 | **0.016** | 0.098 | 1.000 |
| Global perfusion (mL/min/100 g) | Month 3 vs Day 3 | 10 | Paired t-test | 4.20 | -44.78 to 53.18 | 0.851 | 1.000 | 1.000 |
| Global perfusion (mL/min/100 g) | Month 12 vs Day 3 | 9 | Paired t-test | -6.33 | -54.57 to 41.91 | 0.770 | 1.000 | 1.000 |
| Global perfusion (mL/min/100 g) | Month 12 vs Month 3 | 12 | Paired t-test | -6.00 | -33.66 to 21.66 | 0.642 | 1.000 | 1.000 |
| Cortex perfusion (mL/min/100 g) | Day 3 vs Pre-donation | 9 | Paired t-test | -18.44 | -58.09 to 21.20 | 0.315 | 1.000 | 1.000 |
| Cortex perfusion (mL/min/100 g) | Month 3 vs Pre-donation | 10 | Paired t-test | -1.70 | -40.39 to 36.99 | 0.923 | 1.000 | 1.000 |
| Cortex perfusion (mL/min/100 g) | Month 12 vs Pre-donation | 9 | Paired t-test | -26.44 | -63.57 to 10.69 | 0.139 | 0.835 | 1.000 |
| Cortex perfusion (mL/min/100 g) | Month 3 vs Day 3 | 9 | Paired t-test | 28.00 | 5.01 to 50.99 | **0.023** | 0.137 | 1.000 |
| Cortex perfusion (mL/min/100 g) | Month 12 vs Day 3 | 8 | Paired t-test | 1.75 | -38.38 to 41.88 | 0.921 | 1.000 | 1.000 |
| Cortex perfusion (mL/min/100 g) | Month 12 vs Month 3 | 10 | Paired t-test | -11.60 | -39.43 to 16.23 | 0.370 | 1.000 | 1.000 |
| Resistive index | Day 3 vs Pre-donation | 9 | Paired t-test | -0.008 | -0.052 to 0.036 | 0.695 | 1.000 | 1.000 |
| Resistive index | Month 3 vs Pre-donation | 12 | Paired t-test | -0.032 | -0.078 to 0.013 | 0.140 | 0.842 | 1.000 |
| Resistive index | Month 12 vs Pre-donation | 11 | Paired t-test | -0.031 | -0.071 to 0.009 | 0.117 | 0.702 | 1.000 |
| Resistive index | Month 3 vs Day 3 | 10 | Paired t-test | -0.038 | -0.081 to 0.005 | 0.079 | 0.475 | 1.000 |
| Resistive index | Month 12 vs Day 3 | 9 | Paired t-test | -0.033 | -0.069 to 0.002 | 0.061 | 0.364 | 1.000 |
| Resistive index | Month 12 vs Month 3 | 12 | Paired t-test | 0.003 | -0.036 to 0.043 | 0.855 | 1.000 | 1.000 |
| Cortex R2* (1/s) | Day 3 vs Pre-donation | 11 | Wilcoxon | -0.58 | -0.670 to -0.300 | **0.042** | 0.252 | 1.000 |
| Cortex R2* (1/s) | Month 3 vs Pre-donation | 13 | Paired t-test | -0.10 | -0.50 to 0.292 | 0.578 | 1.000 | 1.000 |
| Cortex R2* (1/s) | Month 12 vs Pre-donation | 12 | Paired t-test | -0.15 | -0.57 to 0.28 | 0.468 | 1.000 | 1.000 |
| Cortex R2* (1/s) | Month 3 vs Day 3 | 11 | Paired t-test | 0.39 | -0.021 to 0.80 | 0.061 | 0.363 | 1.000 |
| Cortex R2* (1/s) | Month 12 vs Day 3 | 10 | Paired t-test | 0.35 | -0.21 to 0.91 | 0.187 | 1.000 | 1.000 |
| Cortex R2* (1/s) | Month 12 vs Month 3 | 12 | Paired t-test | -0.090 | -0.54 to 0.36 | 0.666 | 1.000 | 1.000 |
| Medulla R2* (1/s) | Day 3 vs Pre-donation | 11 | Paired t-test | -2.80 | -5.72 to 0.12 | 0.059 | 0.351 | 1.000 |
| Medulla R2* (1/s) | Month 3 vs Pre-donation | 12 | Paired t-test | -2.5 | -4.32 to -0.67 | **0.012** | 0.071 | 0.816 |
| Medulla R2* (1/s) | Month 12 vs Pre-donation | 12 | Paired t-test | -2.29 | -4.16 to -0.43 | **0.021** | 0.123 | 1.000 |
| Medulla R2* (1/s) | Month 3 vs Day 3 | 10 | Paired t-test | 0.55 | -1.19 to 2.28 | 0.493 | 1.000 | 1.000 |
| Medulla R2* (1/s) | Month 12 vs Day 3 | 10 | Paired t-test | -0.47 | -2.15 to 1.22 | 0.544 | 1.000 | 1.000 |
| Medulla R2* (1/s) | Month 12 vs Month 3 | 11 | Paired t-test | -0.19 | -1.35 to 0.96 | 0.719 | 1.000 | 1.000 |
| Cortex ADC (10^-3^ mm^2^/s) | Day 3 vs Pre-donation | 12 | Wilcoxon | 0.13 | 0.085 to 0.16 | **<0.001** | **0.003** | **0.034** |
| Cortex ADC (10^-3^ mm^2^/s) | Month 3 vs Pre-donation | 13 | Paired t-test | 0.063 | -0.025 to 0.15 | 0.146 | 0.876 | 1.000 |
| Cortex ADC (10^-3^ mm^2^/s) | Month 12 vs Pre-donation | 12 | Paired t-test | 0.037 | -0.051 to 0.12 | 0.377 | 1.000 | 1.000 |
| Cortex ADC (10^-3^ mm^2^/s) | Month 3 vs Day 3 | 12 | Paired t-test | -0.073 | -0.15 to -0.001 | **0.047** | 0.283 | 1.000 |
| Cortex ADC (10^-3^ mm^2^/s) | Month 12 vs Day 3 | 11 | Paired t-test | -0.11 | -0.18 to -0.050 | **0.003** | **0.016** | 0.181 |
| Cortex ADC (10^-3^ mm^2^/s) | Month 12 vs Month 3 | 12 | Wilcoxon | -0.070 | -0.11 to 0.00 | 0.396 | 1.000 | 1.000 |

| **Variable** | **Time point** | **n** | **Test** | **Change** | **95% CI** | **p value** | **Bonferroni p value** | **Global Bonferroni p value** |
| --- | --- | --- | --- | --- | --- | --- | --- | --- |
| Medulla ADC (10^-3^ mm^2^/s) | Day 3 vs Pre-donation | 12 | Paired t-test | 0.13 | 0.067 to 0.19 | **<0.001** | **0.005** | 0.056 |
| Medulla ADC (10^-3^ mm^2^/s) | Month 3 vs Pre-donation | 13 | Paired t-test | 0.080 | 0.010 to 0.15 | **0.029** | 0.171 | 1.000 |
| Medulla ADC (10^-3^ mm^2^/s) | Month 12 vs Pre-donation | 11 | Paired t-test | 0.060 | -0.002 to 0.12 | 0.057 | 0.345 | 1.000 |
| Medulla ADC (10^-3^ mm^2^/s) | Month 3 vs Day 3 | 12 | Paired t-test | -0.047 | -0.13 to 0.037 | 0.246 | 1.000 | 1.000 |
| Medulla ADC (10^-3^ mm^2^/s) | Month 12 vs Day 3 | 10 | Paired t-test | -0.070 | -0.16 to 0.018 | 0.106 | 0.637 | 1.000 |
| Medulla ADC (10^-3^ mm^2^/s) | Month 12 vs Month 3 | 11 | Paired t-test | -0.034 | -0.11 to 0.039 | 0.324 | 1.000 | 1.000 |
| Cortex T1 (ms) | Day 3 vs Pre-donation | 8 | Paired t-test | 85.88 | 27.12 to 144.6 | **0.011** | 0.064 | 0.732 |
| Cortex T1 (ms) | Month 3 vs Pre-donation | 10 | Paired t-test | -0.50 | -29.25 to 28.25 | 0.969 | 1.000 | 1.000 |
| Cortex T1 (ms) | Month 12 vs Pre-donation | 9 | Paired t-test | -27.00 | -84.60 to 30.60 | 0.311 | 1.000 | 1.000 |
| Cortex T1 (ms) | Month 3 vs Day 3 | 8 | Wilcoxon | -54.50 | -99.00 to -35.00 | **0.008** | **0.047** | 0.539 |
| Cortex T1 (ms) | Month 12 vs Day 3 | 7 | Paired t-test | -91.14 | -150.5 to -31.74 | **0.009** | 0.057 | 0.653 |
| Cortex T1 (ms) | Month 12 vs Month 3 | 10 | Paired t-test | -31.90 | -75.34 to 11.54 | 0.131 | 0.786 | 1.000 |
| Medulla T1 (ms) | Day 3 vs Pre-donation | 9 | Paired t-test | 140.3 | 1.67 to 279.0 | **0.048** | 0.287 | 1.000 |
| Medulla T1 (ms) | Month 3 vs Pre-donation | 10 | Paired t-test | 46.40 | -35.06 to 127.9 | 0.230 | 1.000 | 1.000 |
| Medulla T1 (ms) | Month 12 vs Pre-donation | 9 | Paired t-test | 103.6 | 52.27 to 154.8 | **0.002** | **0.010** | 0.113 |
| Medulla T1 (ms) | Month 3 vs Day 3 | 9 | Paired t-test | -81.33 | -226.1 to 63.42 | 0.231 | 1.000 | 1.000 |
| Medulla T1 (ms) | Month 12 vs Day 3 | 8 | Paired t-test | -46.00 | -213.1 to 121.1 | 0.536 | 1.000 | 1.000 |
| Medulla T1 (ms) | Month 12 vs Month 3 | 10 | Wilcoxon | 6.50 | -30.50 to 69.00 | 0.695 | 1.000 | 1.000 |

Differences are later minus earlier time point. Change is the mean paired difference for paired t-tests and the median paired difference for Wilcoxon signed-rank tests. Confidence intervals are not adjusted for multiplicity. Tests are two-sided. Bonferroni p = min (p × m, 1), where m = 3 for mGFR and 6 for each MRI variable. Global Bonferroni p = min (p × 69, 1), across all comparisons in this table. Bold indicates p < 0.05 in the respective column. Pre-donation mGFR refers to calculated single-kidney GFR; postoperative mGFR was measured by iohexol clearance. n denotes the number of paired observations.

**TABLE S4**

**Univariable linear regression analysis of factors associated with 12‑month kidney function (mGFR)**

| Variable | Β (95% CI) | p-value | Adj. R2 |
| --- | --- | --- | --- |
| mGFRabs (mL/min) | 0.44 (-0.099 – 0.98) | 0.099 | 0.17 |
| Sk mGFR0abs (mL/min) | 1.24 (0.59 – 1.89) | **0.002** | 0.61 |
| Age (years) | -0.50 (-1.18 – 0.17) | 0.13 | 0.14 |
| Weight (kg) | 0.71 (0.15 – 1.27) | **0.018** | 0.39 |
| Height (m) | 0.74 (-0.060 – 1.54) | 0.066 | 0.23 |
| BSA (m^2^) | 49.12 (13.4 – 84.8) | **0.012** | 0.43 |
| Kidney volume pre-donation (mL) | 0.29 (0.088 – 0.48) | **0.009** | 0.46 |
| Kidney volume day 3 post-donation (mL) | 0.34 (0.10 – 0.57) | **0.010** | 0.49 |
| Kidney volume month 3 post-donation (mL) | 0.30 (0.12 – 0.48) | **0.003** | 0.55 |
| ∆ Kidney volume pre-donation to day 3 (mL) | -0.14 (-0.79 – 0.50) | 0.63 | -0.081 |
| ∆ Kidney volume pre-donation to month 3 (mL) | 0.17 (-0.48 – 0.82) | 0.57 | -0.064 |
| Cortex ADC day 3 (10^-3^ mm^2^/s) | -20.44 (-95.86 – 54.97) | 0.56 | -0.067 |
| ∆ Cortex ADC pre-don to day 3 (10^-3^ mm^2^/s) | -5.97 (-16.14 – 4.75) | 0.24 | 0.061 |
| Medullary ADC day 3 (10^-3^ mm^2^/s) | -34.6 (-88.27 – 19.05) | 0.18 | 0.10 |
| ∆ Medullary ADC pre-don to day 3 | -28.69 (-127.6 – 70.2) | 0.53 | -0.060 |
| Cortex T1 3 days (ms) | 0.048 (-0.098 – 0.19) | 0.45 | -0.052 |
| ∆ Cortex T1 pre-don to day 3 (ms) | -0.039 (-0.14 – 0.065) | 0.39 | -0.020 |
| R2* Medulla day 3 | -0.20 (-1.98 – 1.58) | 0.81 | -0.012 |
| ∆ MedullaR2* pre-don to day 3 | -0.90 (-2.58 – 0.77) | 0.25 | 0.057 |
|  |  |  |  |

**TABLE S5 Bonferroni-corrected linear-mixed model (LMM) pairwise comparisons**

| **Variable** | **Time point** | **Change** | **95% CI** | **p value** | **Bonferroni p value** | **Global Bonferroni p value** |
| --- | --- | --- | --- | --- | --- | --- |
| mGFR (mL/min) | Month 3 vs Pre (single kidney) | 13.9 | 10.9 to 16.8 | **<0.001** | **<0.001** | **<0.001** |
| mGFR (mL/min) | Month 12 vs Pre (single kidney) | 16.9 | 14.0 to 19.8 | **<0.001** | **<0.001** | **<0.001** |
| mGFR (mL/min) | Month 12 vs Month 3 | 3.0 | 0.1 to 5.9 | **0.039** | 0.118 | 1.000 |
| Kidney volume (mL) | Day 3 vs Pre-donation | 31.2 | 25.2 to 37.2 | **<0.001** | **<0.001** | **<0.001** |
| Kidney volume (mL) | Month 3 vs Pre-donation | 26.4 | 21.2 to 31.6 | **<0.001** | **<0.001** | **<0.001** |
| Kidney volume (mL) | Month 12 vs Pre-donation | 34.5 | 27.6 to 41.5 | **<0.001** | **<0.001** | **<0.001** |
| Kidney volume (mL) | Month 3 vs Day 3 | -4.8 | -10.0 to 0.3 | 0.066 | 0.398 | 1.000 |
| Kidney volume (mL) | Month 12 vs Day 3 | 3.3 | -3.4 to 10.0 | 0.330 | 1.000 | 1.000 |
| Kidney volume (mL) | Month 12 vs Month 3 | 8.2 | 2.5 to 13.8 | **0.005** | **0.028** | 0.326 |
| Blood flow (mL/min) | Day 3 vs Pre-donation | 61.7 | 26.0 to 97.4 | **<0.001** | **0.004** | **0.049** |
| Blood flow (mL/min) | Month 3 vs Pre-donation | 62.3 | 11.1 to 113.6 | **0.017** | 0.103 | 1.000 |
| Blood flow (mL/min) | Month 12 vs Pre-donation | 77.4 | 32.0 to 122.8 | **<0.001** | **0.005** | 0.057 |
| Blood flow (mL/min) | Month 3 vs Day 3 | 0.6 | -64.5 to 65.8 | 0.985 | 1.000 | 1.000 |
| Blood flow (mL/min) | Month 12 vs Day 3 | 15.7 | -44.5 to 75.9 | 0.609 | 1.000 | 1.000 |
| Blood flow (mL/min) | Month 12 vs Month 3 | 15.1 | -22.6 to 52.7 | 0.432 | 1.000 | 1.000 |
| Global perfusion (mL/min/100 g) | Day 3 vs Pre-donation | -48.3 | -74.2 to -22.3 | **<0.001** | **0.002** | **0.019** |
| Global perfusion (mL/min/100 g) | Month 3 vs Pre-donation | -35.8 | -68.6 to -3.0 | **0.032** | 0.193 | 1.000 |
| Global perfusion (mL/min/100 g) | Month 12 vs Pre-donation | -42.1 | -69.0 to -15.2 | **0.002** | **0.013** | 0.150 |
| Global perfusion (mL/min/100 g) | Month 3 vs Day 3 | 12.4 | -23.3 to 48.2 | 0.496 | 1.000 | 1.000 |
| Global perfusion (mL/min/100 g) | Month 12 vs Day 3 | 6.2 | -29.1 to 41.4 | 0.732 | 1.000 | 1.000 |
| Global perfusion (mL/min/100 g) | Month 12 vs Month 3 | -6.3 | -29.1 to 16.6 | 0.590 | 1.000 | 1.000 |
| Cortex perfusion | Day 3 vs Pre-donation | -52.9 | -103.7 to -2.1 | **0.041** | 0.248 | 1.000 |
| Cortex perfusion | Month 3 vs Pre-donation | -10.3 | -45.1 to 24.4 | 0.559 | 1.000 | 1.000 |
| Cortex perfusion | Month 12 vs Pre-donation | -23.7 | -51.2 to 3.9 | 0.092 | 0.553 | 1.000 |
| Cortex perfusion | Month 3 vs Day 3 | 42.5 | 18.0 to 67.0 | **<0.001** | **0.004** | **0.046** |
| Cortex perfusion | Month 12 vs Day 3 | 29.2 | -13.2 to 71.6 | 0.177 | 1.000 | 1.000 |
| Cortex perfusion | Month 12 vs Month 3 | -13.3 | -35.0 to 8.3 | 0.228 | 1.000 | 1.000 |
| Resistance index | Day 3 vs Pre-donation | 0.0 | -0.03 to 0.03 | 0.923 | 1.000 | 1.000 |
| Resistance index | Month 3 vs Pre-donation | -0.0 | -0.07 to 0.00 | 0.061 | 0.364 | 1.000 |
| Resistance index | Month 12 vs Pre-donation | -0.0 | -0.06 to 0.00 | **0.028** | 0.168 | 1.000 |
| Resistance index | Month 3 vs Day 3 | -0.0 | -0.07 to -0.01 | **0.015** | 0.089 | 1.000 |
| Resistance index | Month 12 vs Day 3 | -0.0 | -0.06 to -0.01 | **0.006** | **0.034** | 0.393 |
| Resistance index | Month 12 vs Month 3 | 0.0 | -0.03 to 0.03 | 0.932 | 1.000 | 1.000 |
| Cortex R2* (1/s) | Day 3 vs Pre-donation | -0.5 | -0.85 to -0.12 | **0.009** | 0.055 | 0.634 |
| Cortex R2* (1/s) | Month 3 vs Pre-donation | -0.1 | -0.45 to 0.24 | 0.552 | 1.000 | 1.000 |
| Cortex R2* (1/s) | Month 12 vs Pre-donation | -0.2 | -0.52 to 0.19 | 0.357 | 1.000 | 1.000 |
| Cortex R2* (1/s) | Month 3 vs Day 3 | 0.4 | 0.05 to 0.71 | **0.024** | 0.146 | 1.000 |
| Cortex R2* (1/s) | Month 12 vs Day 3 | 0.3 | -0.09 to 0.72 | 0.128 | 0.766 | 1.000 |
| Cortex R2* (1/s) | Month 12 vs Month 3 | -0.1 | -0.43 to 0.31 | 0.739 | 1.000 | 1.000 |
| Medulla R2* (1/s) | Day 3 vs Pre-donation | -2.7 | -4.92 to -0.56 | **0.014** | 0.082 | 0.938 |
| Medulla R2* (1/s) | Month 3 vs Pre-donation | -2.5 | -3.91 to -0.99 | **0.001** | **0.006** | 0.070 |
| Medulla R2* (1/s) | Month 12 vs Pre-donation | -2.7 | -4.36 to -1.01 | **0.002** | **0.010** | 0.116 |
| Medulla R2* (1/s) | Month 3 vs Day 3 | 0.3 | -1.03 to 1.61 | 0.663 | 1.000 | 1.000 |
| Medulla R2* (1/s) | Month 12 vs Day 3 | 0.1 | -1.33 to 1.45 | 0.931 | 1.000 | 1.000 |
| Medulla R2* (1/s) | Month 12 vs Month 3 | -0.2 | -1.15 to 0.69 | 0.621 | 1.000 | 1.000 |
| Cortex ADC (10^-3 mm^2/s) | Day 3 vs Pre-donation | 0.1 | 0.09 to 0.19 | **<0.001** | **<0.001** | **<0.001** |
| Cortex ADC (10^-3 mm^2/s) | Month 3 vs Pre-donation | 0.1 | -0.01 to 0.14 | 0.106 | 0.634 | 1.000 |
| Cortex ADC (10^-3 mm^2/s) | Month 12 vs Pre-donation | 0.0 | -0.04 to 0.10 | 0.355 | 1.000 | 1.000 |
| Cortex ADC (10^-3 mm^2/s) | Month 3 vs Day 3 | -0.1 | -0.14 to -0.02 | **0.010** | 0.057 | 0.660 |
| Cortex ADC (10^-3 mm^2/s) | Month 12 vs Day 3 | -0.1 | -0.16 to -0.05 | **<0.001** | **<0.001** | **0.006** |
| Cortex ADC (10^-3 mm^2/s) | Month 12 vs Month 3 | -0.0 | -0.08 to 0.02 | 0.280 | 1.000 | 1.000 |
| Medulla ADC (10^-3 mm^2/s) | Day 3 vs Pre-donation | 0.1 | 0.07 to 0.18 | **<0.001** | **<0.001** | **<0.001** |
| Medulla ADC (10^-3 mm^2/s) | Month 3 vs Pre-donation | 0.1 | 0.02 to 0.14 | **0.010** | 0.058 | 0.662 |
| Medulla ADC (10^-3 mm^2/s) | Month 12 vs Pre-donation | 0.1 | 0.00 to 0.10 | 0.057 | 0.339 | 1.000 |
| Medulla ADC (10^-3 mm^2/s) | Month 3 vs Day 3 | -0.1 | -0.11 to 0.02 | 0.187 | 1.000 | 1.000 |
| Medulla ADC (10^-3 mm^2/s) | Month 12 vs Day 3 | -0.1 | -0.16 to 0.01 | 0.086 | 0.519 | 1.000 |
| Medulla ADC (10^-3 mm^2/s) | Month 12 vs Month 3 | -0.0 | -0.10 to 0.04 | 0.379 | 1.000 | 1.000 |
| Cortex T1 (ms) | Day 3 vs Pre-donation | 62.3 | 17.7 to 106.9 | **0.006** | **0.037** | 0.426 |
| Cortex T1 (ms) | Month 3 vs Pre-donation | 1.0 | -22.5 to 24.5 | 0.932 | 1.000 | 1.000 |
| Cortex T1 (ms) | Month 12 vs Pre-donation | -22.1 | -68.8 to 24.7 | 0.354 | 1.000 | 1.000 |
| Cortex T1 (ms) | Month 3 vs Day 3 | -61.3 | -100.3 to -22.2 | **0.002** | **0.013** | 0.146 |
| Cortex T1 (ms) | Month 12 vs Day 3 | -84.4 | -134.4 to -34.3 | **<0.001** | **0.006** | 0.066 |
| Cortex T1 (ms) | Month 12 vs Month 3 | -23.1 | -60.6 to 14.4 | 0.227 | 1.000 | 1.000 |
| Medulla T1 (ms) | Day 3 vs Pre-donation | 131.6 | 38.1 to 225.1 | **0.006** | **0.035** | 0.401 |
| Medulla T1 (ms) | Month 3 vs Pre-donation | 87.1 | -10.5 to 184.8 | 0.080 | 0.481 | 1.000 |
| Medulla T1 (ms) | Month 12 vs Pre-donation | 100.9 | 58.2 to 143.5 | **<0.001** | **<0.001** | **<0.001** |
| Medulla T1 (ms) | Month 3 vs Day 3 | -44.4 | -172.1 to 83.2 | 0.495 | 1.000 | 1.000 |
| Medulla T1 (ms) | Month 12 vs Day 3 | -30.7 | -127.4 to 65.9 | 0.533 | 1.000 | 1.000 |
| Medulla T1 (ms) | Month 12 vs Month 3 | 13.7 | -68.1 to 95.6 | 0.743 | 1.000 | 1.000 |
| Bonferroni p = min (p × m, 1), where m = 3 for mGFR and 6 for each MRI variable. Global Bonferroni p = min (p × 69, 1), across all comparisons in this table. Bold indicates p < 0.05 in the respective column. Pre-donation mGFR refers to calculated single-kidney GFR; postoperative mGFR was measured by iohexol clearance. | | | | | | |

**TABLE S6**

**Multivariable linear regression models for factors associated with 12-month mGFR**

| **Model** (*cortical ADC, n=11*) |  |  |  |
| --- | --- | --- | --- |

| **Variable** | **B (95% CI)** | **p value** | **Adjusted R²** |
| --- | --- | --- | --- |
| Kidney volume at month 3 (mL) | 0.36 (0.18–0.55) | **0.002** | 0.67 |
| Δ Cortical ADC (pre-donation to day 3) (10⁻³ mm²/s) | −4.04 (−44.38–36.29) | 0.823 |  |

**TABLE S7**

**Compact diagnostics for the exploratory multivariable regression model with 12-month measured GFR as outcome.**

| **Predictor** | **Estimate** | **95% CI** | **p value** | **VIF** | **Leave-one-out estimate range** | **Leave-one-out p-value range** | **Sensitivity interpretation** |
| --- | --- | --- | --- | --- | --- | --- | --- |
| 3-month kidney volume (mL) | 0.386 | 0.249 to 0.524 | **<0.001** | 1.018 | 0.346 to 0.432 | <0.001 to 0.001 | Significant in all leave-one-out iterations |
| Medullary ADC change day 3 minus pre-donation (10^-3^ mm^2^/s) | -44.928 | -88.093 to -1.763 | **0.043** | 1.018 | -58.164 to  -28.574 | 0.003 to 0.178 | Negative estimate in all iterations; significance varied |

VIF; variance inflation factor. Leave-one-out ranges show the range of model estimates and p values after excluding one participant at a time. The medullary ADC variable was calculated from raw values as postoperative day 3 minus pre-donation and is expressed in 10⁻³ mm²/s.
